# Supplementary material for: Imprinting of Mesenchymal Stromal Cell Transcriptome Persists even after Treatment in Patients with Multiple Myeloma
Source: Int J Mol Sci. 2020 May 28;21(11):3854. doi: 10.3390/ijms21113854 (PMC7312921; doi:10.3390/ijms21113854)
Supplement: Supplementary file 1 [file ijms-21-03854-s001.pdf]

Supplemental data

Material and methods

Flow cytometry antibody and staining

MSC and PBMC were stained with different combinations of monoclonal antibody anti-CD14-BUV395 (BD Biosciences), -CD138-BV421 (BD Biosciences), -CD11b-BV605 (BD Biosciences), -CD90-PerCpCy5.5 (BD Biosciences), -CD105-PE (eBioscience), -CD38-PECy7 (BD Biosciences), -CD73-APC (BD Biosciences), -CD45-AF700 (BD Biosciences), -viability-BV510 (BioLegend), -CD8-BUV395 (BD Biosciences), -CD3-BV711 (BD Biosciences), -CD4-FITC (BD Biosciences), -CD45-PE (BD Biosciences), fixable viability Dye eFluor 780 (ThermoFisher) and used at dilutions according to manufacturers' instructions. Then they were acquired with a Fortessa X20 flow cytometer (BD Biosciences) and analyzed with FlowJo Software (TreeStar).

Table 1

Gene	Assay ID
GAPDH	Hs02786624_g1
SP7	Hs01866874_s1
RUNX2	Hs01047973_m1
ALPL	Hs01029144_m1
PTHR1	Hs00174895_m1
IBSP	Hs00173720_m1
PPARg	Hs00234592_m1
AdipoQ	Hs00605917_m1
LEP	Hs00174877_m1

Figure legends

Figure S1: Cytometry was performed with MM and HD BM-MSC culture and shows 99.9% cells negative for the hematopoietic lineage (CD45-), monocyte-negative (CD14-, CD11b-), B cell-negative (CD20), plasma cell-negative (CD138-) and positive for MSC

markers CD73+, CD90+, CD105+ **(A)**. Histogram of the FMO control compared to HD (blue) and MM (orange) MSC-positive markers CD73, CD90 and CD105 **(B)**.

Figure S2: Coagulation pathway involved in difference between MM BM-MSC and HD BM-MSC

Volcano plot of differentially expressed genes in MM vs. HD BM-MSC with impact of genes on coagulation pathway **(A)**. Representative GSEA of MM vs. HD BM-MSC list of expressed genes with pathway Gene Set 28 (Hallmark coagulation). Coagulation is upregulated by MM vs. HD BM-MSC. The corresponding heatmap of represented genes is also shown **(B)**.

Figure S3: Transcriptional profile of D BM-MSC and CR BM-MSC from the same patient

Unsupervised gene expression analysis of D BM-MSC (n=1) and CR BM-MSC (n=1) representing a heatmap with under expression in green and overexpression in pink. No significant differences were observed between D BM-MSC and CR BM-MSC after MM treatment.

Figure S4: Volcano plot of differentially expressed genes in MM vs. HD BM-MSC with impact of immune checkpoint genes

Representative genes of MM vs. HD BM-MSC list of ligand of immune checkpoint genes.

Figure S1

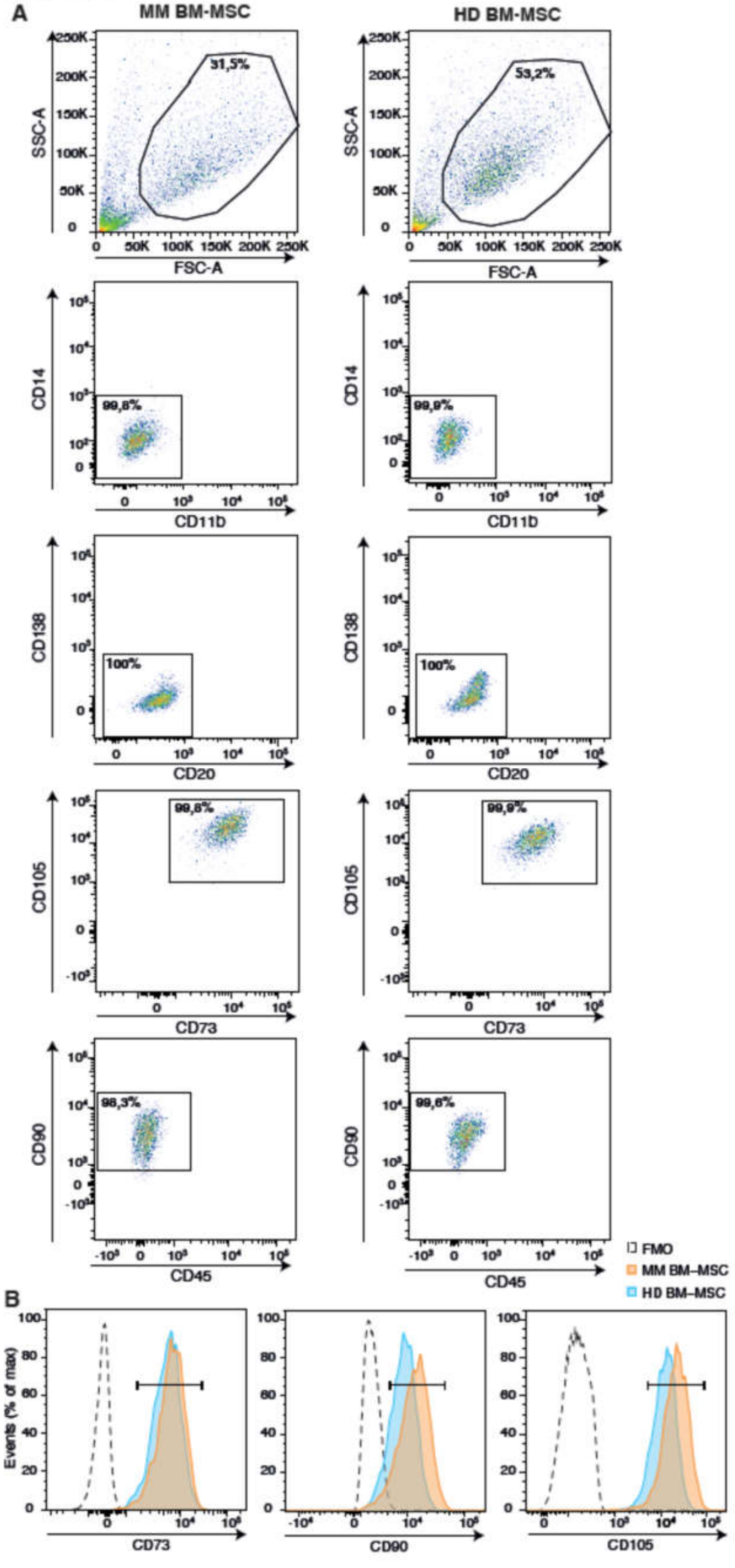
A
MM BM-MSC
HD BM-MSC
31,5%
53,2%
SSC-A
FSC-A
99,8%
99,9%
CD14
CD11b
100%
CD138
CD20
99,8%
99,9%
CD105
CD73
98,3%
99,8%
CD90
CD45
FMO
MM BM–MSC
HD BM–MSC
B
Events (% of max)
CD73
CD90
CD105

Figure S2

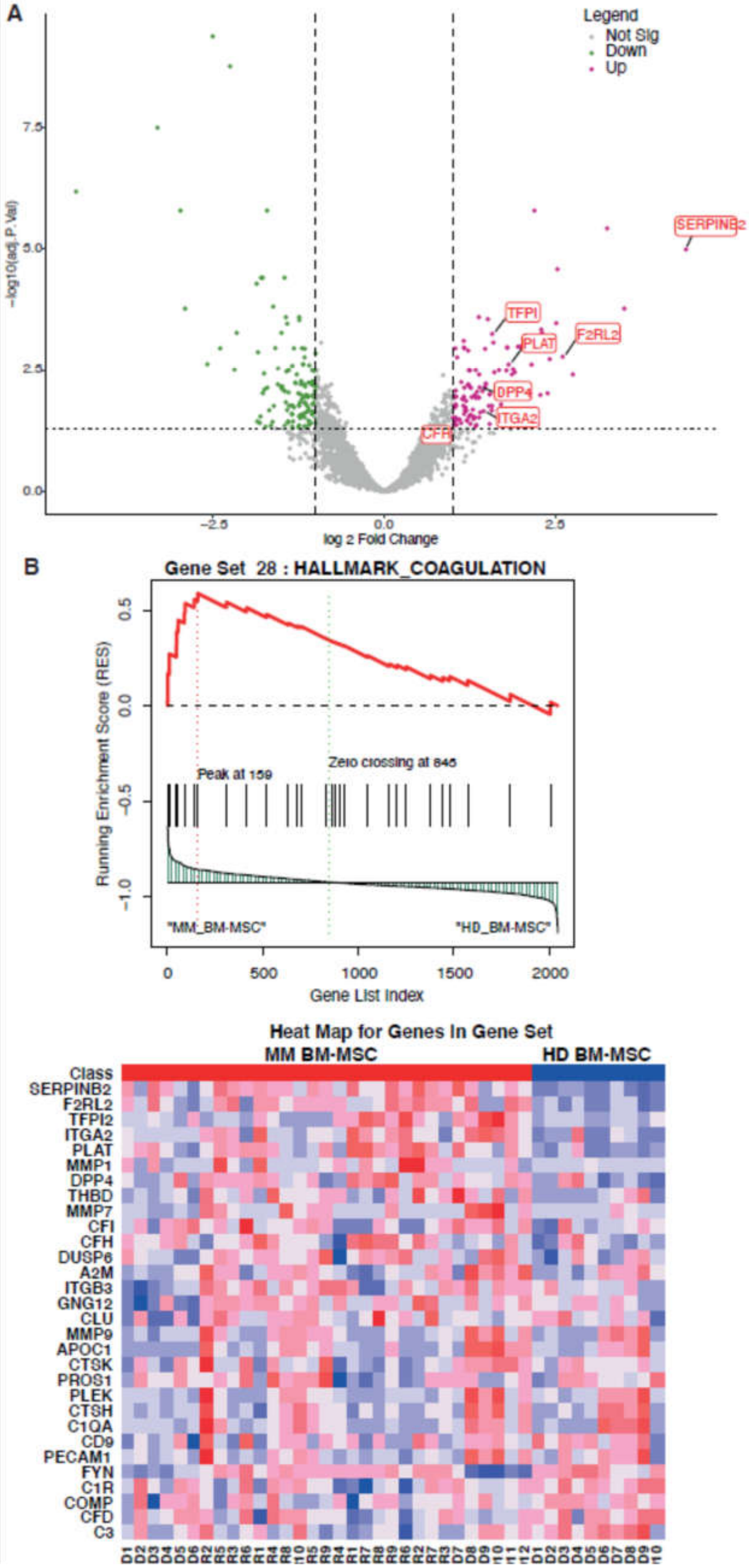
A
Legend
Not Sig
Down
Up
SERPINB2
TFPI
PLAT
F2RL2
DPP4
ITGA2
CFH
−log10(adj. P. Val)
log 2 Fold Change
B
Gene Set 28 : HALLMARK_COAGULATION
Running Enrichment Score (RES)
Peak at 159
Zero crossing at 846
"MM_BM-MSC"
"HD_BM-MSC"
Gene List Index
Heat Map for Genes In Gene Set
MM BM-MSC
HD BM-MSC
Class
SERPINB2
F2RL2
TFPI2
ITGA2
PLAT
MMP1
DPP4
THBD
MMP7
CFI
CFH
DUSP6
A2M
ITGB3
GNG12
CLU
MMP9
APOC1
CTSK
PROS1
PLEK
CTSH
C1QA
CD9
PECAM1
FYN
C1R
COMP
CFD
C3

Figure S3

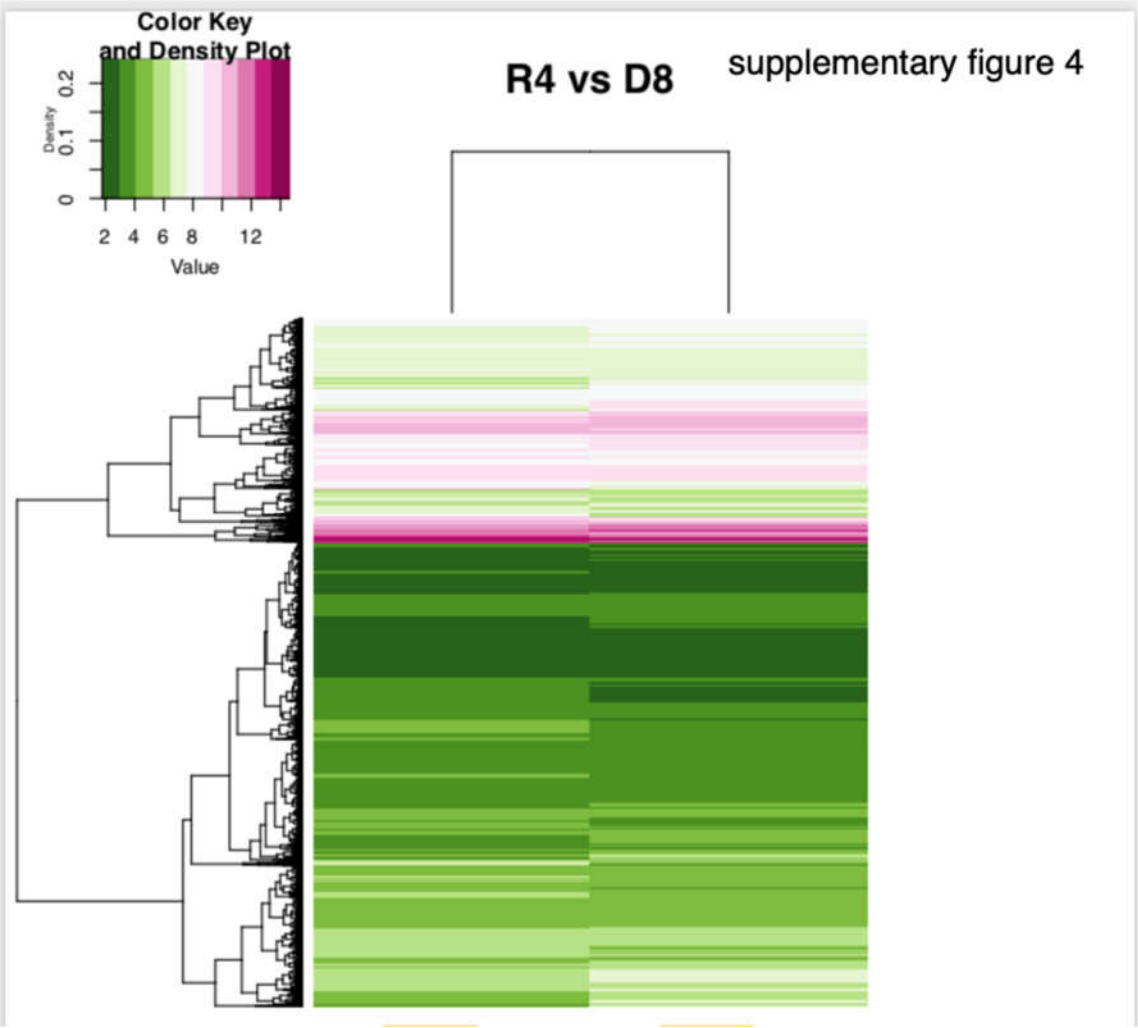
Color Key
and Density Plot
Density
0
0.1
0.2
2
4
6
8
12
Value
R4 vs D8
supplementary figure 4

Figure S4

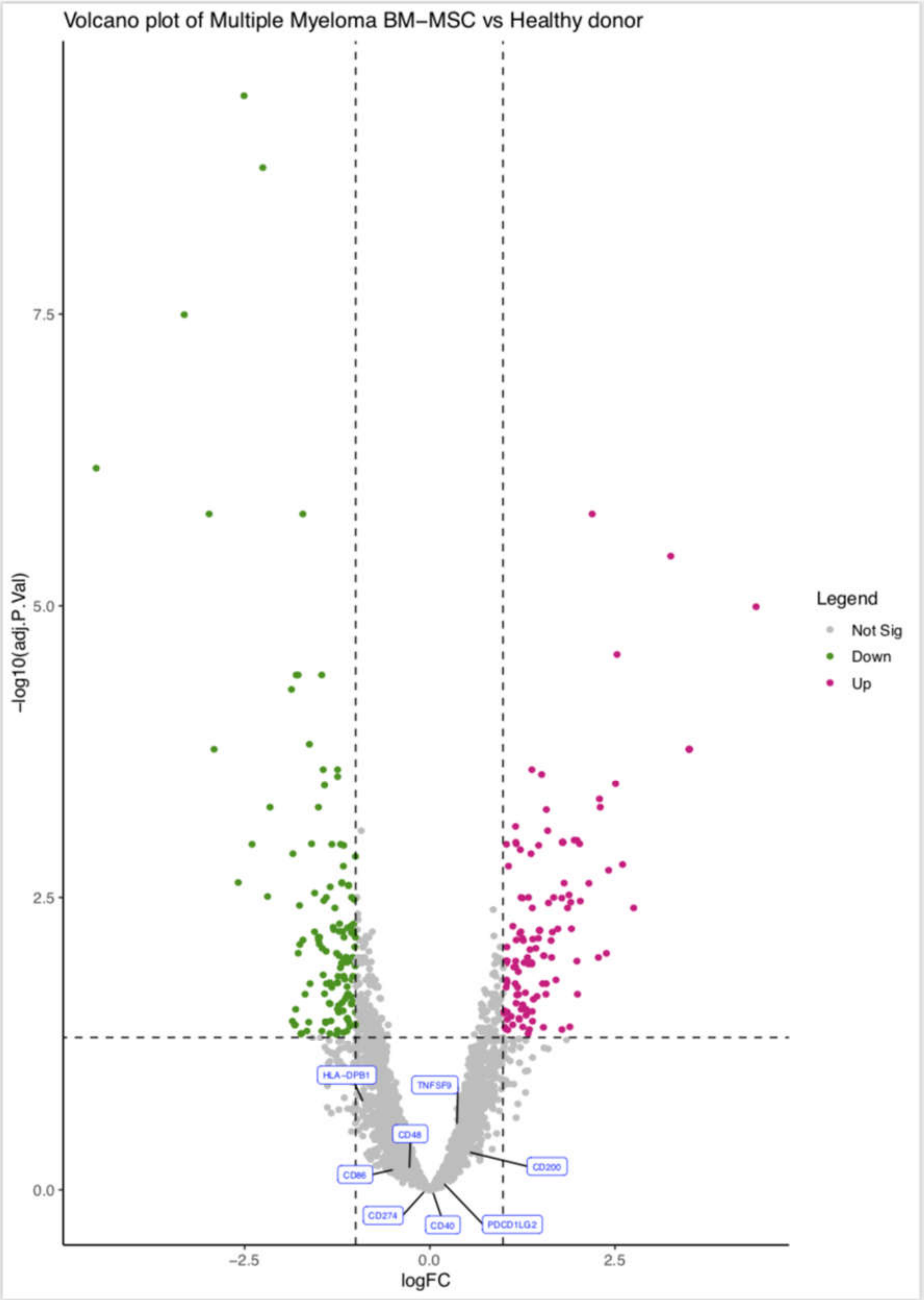
Volcano plot of Multiple Myeloma BM−MSC vs Healthy donor
−log10(adj.P.Val)
7.5
5.0
2.5
0.0
−2.5
0.0
2.5
logFC
Legend
Not Sig
Down
Up
HLA−DPB1
TNFSF9
CD48
CD86
CD200
CD274
CD40
PDCD1LG2
